# A Case of Accidental Swallowing of a Cent of the Old Coinage

**Published:** 1857-10

**Authors:** P. H. Strong

**Affiliations:** Buffalo


ART. III.—*A Case of Accidental Swallowing of a Cent of the old coinage.* Communicated to the Buffalo Medical Association. By P. H. STRONG, M. D., Buffalo.

I send you herewith, according to promise, an account from my notes of the case of accidental swallowing, of which I gave an oral and somewhat indefinite statement from memory, at the last meeting of the association It is one that I had intended to furnish the association ere now. The discussion springing up unanticipated, I was unprepared that evening to give a precise and accurate statement of the case.

Considering the tenderness of the age of the child, with the dimensions of the foreign body, it is believed to constitute a kind of climax in hazardous and yet successful swallowing. I have found nothing (after considerable search) in the records of medicine that quite equals it. Being thus far, perhaps, the ultimate fact of actual achievement in this direction, and, as such, desirable as a matter of reference on the occurrence of like cases, it seems demanded that a detailed and accurate account of it should be placed on record.

Dec. 15, 1856, I was called in great haste to see A. B., aged 2 years and 10 months, son of P. B., Palmer street, in this city, supposed to have swallowed a cent, of the old coinage.

The child was much less than of medium size and vigor of constitution.

He had been considered as quite the most delicate in physical development of any of a family of eight or nine children.

I found the child lying languid, for the most part quiet and unexcited, but occasionally retching with moderate severity, able to swallow with facility, though not desiring, liquids. On asking for the evidence that he had swallowed the cent, I was told by the mother, that he was playing with one by himself and a little one side from the rest of the family; that he was seen momentarily to choke and struggle, and upon their running to him and asking him what he had done with the cent, he (not being able to talk much) pointed into his mouth and throat, indicating that he had swallowed it.

Supposing it a physical impossibility, and deeming the evidence, in his appearance, and from their account, not quite conclusive, and moreover there being no symptoms sufficiently urgent, in my judgment, to demand the introduction of any detective instrument into the æsophagus, I contented myself with merely a digital examination of the cervical portion of that tube. This gave no indications of any thing foreign being lodged there, and I concluded that if swallowed (which seemed dubious) it had passed into the stomach. I prescribed quietude with a free use of albumen of eggs to guard against the possible contingency of poisoning; and held further opinions and action in abeyance for further developments.

The next day the child appeared much the same. Quiet, painless, disinclined to exertion; retching continuing, though not so frequent; some thirst, no appetite; slight febrile movement; unable or unwilling to attempt, to swallow food subjected to mastication, but both willing and able to swallow liquid nourishment, and retaining it afterward for the most part.

I waited upon very much this train of symptoms till the 20th, my incredulity the while gradually giving way. I became pretty well satisfied by that date that the child had actually swallowed the cent, and that it remained lodged in the stomach, though neither during this interval, nor at *any* time, did he give any indications of suffering from any toxicological properties of the copper, farther than the nausea and retching which were *most* evident the first two or three days.

Upon the 20th the child appeared somewhat more restless, with considerable loss of vital energy and strength, and increased difficulty in deglutition, even of liquids, and I determined to explore with the *probang*. The instrument was arrested in the region parallel to the lower margin of the thyroid cartilage, and it required considerable and steadily sustained pushing force from this point to a point estimated to be nearly parallel to the lower portion of the sternum, when the resistance gave way, and as I supposed, the obstructing body passed into the stomach.

Immediately after the operation, the child took quite a piece of dry bread and chewed and swallowed it with avidity, and retained it, which confirmed me in the opinion that the coin was dislodged from the æsophagus.

The retching, however, was resumed soon after, and continued with occasioned vomiting, for about a week longer, after which the retching and vomiting were less urgent. The child, however, continued with loss of appetite and in an unthriving emaciating way, till the 12th of Feb., four weeks from the accident, when the cent passed per anum.

All untoward symptoms very soon disappeared, and the child's recovery was complete.

I regret exceedingly that I have not the trophy to present to the association. It was kept for me, and sent to my office, as I had enjoined, but the little girl who brought it, not finding me in, took it back home. It was laid up subject to my call, but upon my calling for it soon thereafter, it could not be found. Suspicion fastened strongly on the grog-loving father, whose bibulous proclivities are supposed to have overmastered all his zeal for facts for scientific purposes, and thus the otherwise famous coin was devoted, for the second time, to an ignoble end.

This report concerning it, however, was and is entirely reliable, and that was that the cent had undergone no change except it was blackened, indicating a superficial oxidation.

The cent of the old coinage is, I believe, nearly pure copper, fourteen lines in diameter. and two lines in thickness, and weighs 167 grains.

The treatment after pushing the coin into the stomach, was to wait upon the unaided and undisturbed powers of nature, which I believe is the advice of the best authorities, with foreign bodies of the shape and size of this.

Instances are abundant in which older children by a year or two and more, have swallowed the cent, and its equivalent in size, of other bodies, and also others as young and even of more tender age, have swallowed the dime, the new cent, and other coins and buttons of about the same size; but none of which I am aware have been quite so extraordinary. In being called to like cases where distress and anxiety is so great and pervading, I believe it is the common experience of the profession, that next to the pleasure of removing at once and completely the offending body, is the comfort and satisfaction of being able to refer to authentic records of successful results in cases of as bad or even of worse character, when prompt extraction or removal was precluded. In the hope that the present case may at least serve some one this *second* grade of satisfaction and profit, I now submit it to the association.

P. H. STRONG.

BUFFALO, Sept., 1857.

